# The Rise of the CRISPR/Cpf1 System for Efficient Genome Editing in Plants

**DOI:** 10.3389/fpls.2020.00264

**Published:** 2020-03-31

**Authors:** Anshu Alok, Dulam Sandhya, Phanikanth Jogam, Vandasue Rodrigues, Kaushal K. Bhati, Himanshu Sharma, Jitendra Kumar

**Affiliations:** ^1^University Institute of Engineering and Technology, Panjab University, Chandigarh, India; ^2^Department of Biotechnology, Kakatiya University, Warangal, India; ^3^Copenhagen Plant Science Centre, Department of Plant and Environmental Sciences, University of Copenhagen, Copenhagen, Denmark; ^4^Louvain Institute of Biomolecular Science, UCLouvain, Louvain-la-Neuve, Belgium; ^5^CSK Himachal Pradesh Agricultural University, Palampur, India; ^6^Department of Plant Pathology, University of Minnesota, Minneapolis, MN, United States

**Keywords:** ZFN, TALEN, Cas9, Cpf1, crRNA, CRISPR, NHEJ, endoribonuclease

## Abstract

Cpf1, an endonuclease of the class 2 CRISPR family, fills the gaps that were previously faced in the world of genome engineering tools, which include the TALEN, ZFN, and CRISPR/Cas9. Other simultaneously discovered nucleases were not able to carry out re-engineering at the same region due to the loss of a target site after first-time engineering. Cpf1 acts as a dual nuclease, functioning as an endoribonuclease to process crRNA and endodeoxyribonuclease to cleave target sequences and generate double-stranded breaks. Additionally, Cpf1 allows for multiplexed genome editing, as a single crRNA array transcript can target multiple loci in the genome. The CRISPR/Cpf1 system enables gene deletion, insertion, base editing, and locus tagging in monocot as well as in dicot plants with fewer off-target effects. This tool has been efficiently demonstrated into tobacco, rice, soybean, wheat, etc. This review covers the development and applications of Cpf1 mediated genome editing technology in plants.

## Introduction

The rapid advancement of genetic engineering tools for functional genomics studies has facilitated a deeper understanding of biological processes. This decade is witnessing revolutionary uses of RNA-guided endonucleases for trait improvement and disease resistance in plants by genome editing (Wang et al., 2014; Ma et al., 2018; Macovei et al., 2018). The **C**lustered **R**egularly **I**nterspaced **S**hort **P**alindromic **R**epeats (CRISPR) and its associated effector nucleases from bacteria generally belongs to two classes: Class 1 (requires multiple effector proteins) and Class 2 (requires a single effector protein). These two classes have been further diversified into six subtypes. Among them, subtype II contains Cas9 and subtype V contains the Cpf1 effector (Hille and Charpentier, 2016; Koonin et al., 2017). The natural action of the CRISPR/Cas9 system on the viruses has been mimicked and applied as a genetic engineering tool in different kingdoms, including the plant kingdom (DiCarlo et al., 2013; Hou et al., 2013; Hwang et al., 2013; Upadhyay et al., 2013; Jiang et al., 2017). Various basic research in the area of CRISPR biology within the last four decades have finally resulted in using CRISPR/Cas9 as a genome-editing tool. This tool has been successfully applied in different crops plants, such as *Triticum aestivum* (wheat), *Oryza sativa* (rice), *Zea maize* (maize), *Brassica oleracea*, *Brassica rapa* (mustard), *Solanum lycopersicum* (tomato), and *Solanum tuberosum* (potato) as well as fruit plants, such as *Musa acuminata* (banana), *Malus domestica* (apple), *Citrus X sinensis* (orange), and *Vitis vinifera* L. (grapes) (Jiang et al., 2013; Jia and Wang, 2014; Malnoy et al., 2016; Alok et al., 2017; Shimatani et al., 2017; Kaur et al., 2018; Lee et al., 2019).

Cpf1 endonuclease stands for **C**RISPR from ***P****revotella* and ***F****rancisella*1, which was previously known as Cas12a. The CRISPR/Cpf1 system has recently gained more popularity as a better substitute for CRISPR/Cas9 and an advanced and more efficient version of a genome-editing tool (Bin Moon et al., 2018). Cpf1 endonuclease is small in size compared to Cas9 and requires shorter CRISPR RNA (crRNA) to work properly (Liu et al., 2017). Cpf1 is an effector nuclease protein guided by a single RNA that binds upstream of the protospacer adjacent motif (PAM) and cuts the DNA at the proximal end of the PAM, far away from the seed region, by introducing 5 base pair (bp) staggered cuts (Zetsche et al., 2015). The CRISPR/Cpf1 system does not require trans-activating crRNA (tracrRNA) while processing Cpf1-associated CRISPR repeats into mature crRNAs (Zetsche et al., 2015; Zhang et al., 2017). This system proficiently cleaves target DNA adjacent to a short T-rich PAM; Cas9, on the other hand, works with a G-rich PAM. Various studies have demonstrated the use of Cpf1 nuclease for targeted genome editing in prokaryotes (Jiang et al., 2017) as well as eukaryotes (Zetsche et al., 2015; Kim H. et al., 2017). Different orthologs of Cpf1 nucleases from various bacteria were isolated and assessed for genome editing, including AsCpf1 and LbCpf1, which were isolated from *Acidaminococcus* sp. BV3L6 and *Lachnospiraceae bacterium ND2006*, respectively, and have been extensively used for alterations within a genome (Tóth et al., 2016; Moreno-Mateos et al., 2017; Wang et al., 2017). The main advantage of a CRISPR/Cpf1-mediated genome-editing tool is the reengineering of the desired DNA as the target and that the PAM sequence (5′-TTTN-3′) remains intact. The PAM sequence may vary according to its origin of ortholog. The Cpf1-database^1^ is an online tool that offers a simple and easy way to find a potential target within the genome and design gRNA, and it can recognize AsCpf1 and LbCpf1 nucleases through DNA recognition sequences (Park and Bae, 2017).

In the present review, we have discussed structural organization and different orthologs of the Cpf1 endonuclease protein and their efficacy toward genome editing in plants. We also discussed sequence variation in crRNA and PAM for different orthologs of Cpf1. Additionally, we have explored different types of CRISPR/Cpf1 vectors used for single and multiple gene editing, transcriptional activation, suppression, gene knock-in, and base editing. In addition, we have also assessed the features and limitations of Cpf1.

## Structure of Cpf1 Endonuclease

The Cpf1 endonuclease is a bilobed protein that consists of a helical recognition (REC) lobe; it recognizes a CrRNA-target DNA heteroduplex and a nuclease (NUC) lobe, which cut both strands of DNA. The REC lobe is comprised of two domains; REC1 consists of 13 α helices, whereas REC2 is composed of 10 α helices and two β strands. The NUC lobe is comprised of RuvC, WED, PI, and Nuc domains. The PI domain interacts with PAM, whereas the Nuc domain cleaves the DNA. The RuvC domain is made up of three motifs: RuvC-I, RuvC-II, and RuvC-III (Yamano et al., 2016).

## Orthologs of Cpf1

The lengths of Cpf1 orthologs vary across the bacteria, ranging between ∼1,200 and ∼1,500 amino acids (aa). A sequence analysis of 16 Cpf1 orthologs showed that the 5′ sequence of the direct repeat is much more diverse (Zetsche et al., 2019). The direct repeat sequences of LbCpf1, BpCpf1, and SsCpf1 from *Lachnospiraceae bacterium MC2017*, *Butyrivibrio proteoclasticus*, and *Smithella* sp. *SC_K08D17*, respectively, were different from the *Francisella novicida U112* Cpf1 (FnCpf1) (Zetsche et al., 2015). The Cpf1 from *Acidaminococcus* spp. BV3L6 (AsCpf1), *Lachnospiraceae bacterium* ND2006 (LbCpf1), and FnCpf1 have been efficiently used for genome editing in *Saccharomyces cerevisiae* (Verwaal et al., 2018). The PAM regions of Cpf1 orthologs vary between bacteria to bacteria. For example, the LbCpf1 and AsCpf1 endonucleases require 5′-TTTV-3′ PAM (V is A, G, or C), while FnCpf1 requires 5′-TTN-3′ as PAM site. The size of LbCpf1 is 1,228 aa, which is smaller than AsCpf1 (1,307 aa). In green alga, LbCpf1 has been shown to be more active and efficient in gene editing compared to AsCpf1 (Ferenczi et al., 2017). However, both, LbCpf1 and AsCpf1 are more efficient in their mammalian genome editing than FnCpf1 and MbCpf1 (Tu et al., 2017). Other Cpf1 orthologs isolated from *Thiomicrospira* sp. Xs5 (TsCpf1), *Moraxella bovoculi* AAX08_00205 (Mb2Cpf1), *Moraxella bovoculi* AAX11_00205 (Mb3Cpf1), and *Butyrivibrio* sp. NC3005 (BsCpf1) were also used to achieve the desired genome editing in human cells (Zetsche et al., 2019). Mb3Cpf1-mediated editing of a target sequence that has 5′-TTTV-3′ PAM (where is V may be A, C, or G) is comparable to the use of AsCpf1 and LbCpf1 (Zetsche et al., 2019). In rice, AsCpf1 and LbCpf1 were used for genome editing, which showed a higher editing efficiency by LbCpf1 as compared to AsCpf1 (Tang et al., 2017; Li et al., 2018b). Recently, the editing efficiency of LbCpf1 in allotetraploid cotton was tested for the first time. Approximately, 87% editing efficiency was achieved in T0 cotton plants, which indicates a robust editing efficiency of LbCpf1 in allotetraploid cotton (Li et al., 2019). LbCpf1, also known as Cas12a, was used for genome editing in *Nicotiana benthamiana*, *Solanum lycopersicum*, *Arabidopsis thaliana*, and citrus (Bernabé-Orts et al., 2019; Jia et al., 2019).

## Sequence Variation of crRNA and Pam for Cpf1 Orthologs

Almost all variants and orthologs of Cpf1 require a 43-nucleotide-long crRNA as compared to Cas9, which requires both tracrRNA and crRNA. The crRNA is made up of a 20-nucleotide 5′-handle and a 23-nucleotide leader sequence. The leader sequence consists of a seed region and 3′ termini, both of which are complementary to the target region in the genome (Li et al., 2017). Although, the PAM for different orthologs may vary, most of the Cpf1 nucleases require thymine-rich PAM. Earlier, the target range for genome editing using a Cpf1 endonuclease was restricted due to a limitation with PAM recognition sequences. However, researchers have explored different orthologs and engineered variants of Cpf1 to overcome this limitation, and these are able to identify the alternative PAM. Different studies have demonstrated an increased Cpf1 targeting range using *in vitro* and *in vivo* (*E. coli)* PAM identification assays (Zhang et al., 2017). The two Cpf1 endonucleases, AsCpf1 and LbCpf1, require TTTV as a PAM sequence, where V can be A, C, or G nucleotides. Mutations at position S542R/K607R and S542R/K548V/N552R produced AsCpf1 variants, and these are able to recognize TYCV and TATV PAMs, respectively, where Y can be C or T (Gao et al., 2017). The AsCpf1 showed increased activity for TTTV PAMs and decreased activity with TTTT PAM (Kim H. K. et al., 2017). The effect of crRNA length also has clear effects on mutation frequency by Cpf1 nucleases. The strong editing activity of Cpf1 requires 17 to 19 bp of guide sequences. However, 4–5 bp at the 3′ end of a 23 bp guide sequence are not necessary for DNA break (Kleinstiver et al., 2016). The shortening of five bp from the 3′ end of a 23 bp-long guide sequence reduced the indel frequency (Kim H. K. et al., 2017; Verwaal et al., 2018). The LbCpf1-RR and LbCpf1-RVR variants relaxed to recognize and work at “CCCC,” “TYCV,” and “TATG” PAM sites, respectively (Gao et al., 2017). There was a high success rate in rice protoplast for editing CCCC and TYCV PAM sites using an LbCpf1-RR variant and TATG PAM sites using an LbCpf1-RVR variant (Zhong et al., 2018). FnCpf1 variants, i.e., FnCpf1-RR and FnCpf1-RVR, were used in rice for genome editing (Zhong et al., 2018). The FnCpf1 was initially known to edit TTV PAM sites *in vitro*; however, this did not work in human cells (Zetsche et al., 2015; Tu et al., 2017; Zhong et al., 2018). In plants, only one report showed that FnCpf1 worked for a TTV PAM site (Endo et al., 2016). In rice, FnCpf1 showed activity against a TTV PAM site with VTTV PAM combinations but not against GTTA and GTTC PAM (Zhong et al., 2018).

## CRISPR/Cpf1 Components as an Editing Tool

**FIGURE 1:**
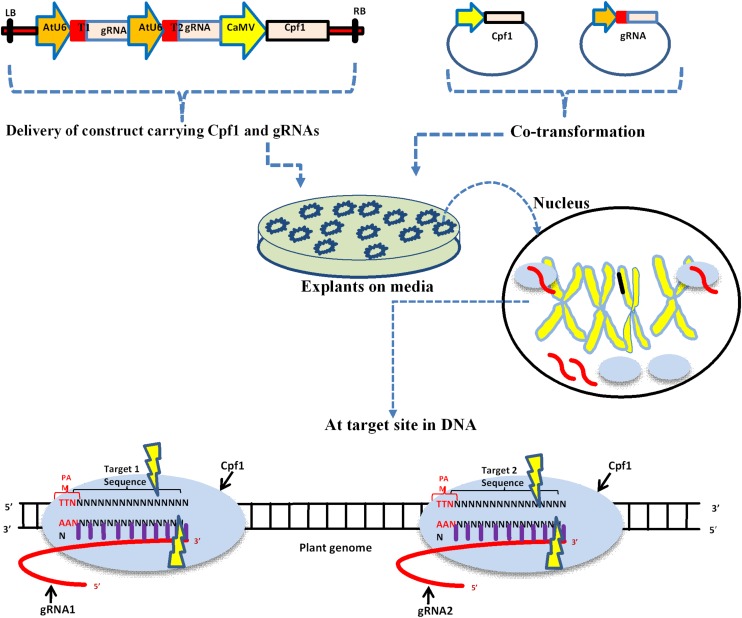
Schematic diagram of delivery of CRISPR/Cpf1 vector into plant cells and the mechanism action of editing. Upper panel depicts single construct and individual vectors carrying Cpf1 and gRNA. LB: left border; T1: Target one; AtU6: Small RNA promoter; CaMV: constitutive promoter, regulates Cpf1; and RB: right border. The delivery of these vectors into plant tissue is usually done by *Agrobacterium*, gene gun, and PEG mediated co-transformation. The black circle represents the nucleus where Cpf1 and gRNA are expressed. The lower panel depicts the mechanism of editing at a target site within DNA.

Genome editing in plants using Cpf1 requires two important components, the Cpf1 protein and a synthetic crRNA. The crRNA fusion with a target DNA with 20 bp results in to a guide RNA. These two components, if they reside on the same vector or two different vectors, can be used for delivery within plant cells (Figure 1). The delivery of the CRISPR/Cpf1 vector into explant is generally carried out using particle bombardment or *Agrobacterium* mediated. Cpf1 recognizes the base pairing between the guide RNA and target DNA within the genome, and the double-stranded breaks are created by the endonuclease activity.

## DeadCpf1 as a Transcriptional Repressor/Activator

Cpf1 has a dual nature related to its activity: it acts as RNase on a modified CRISPR array as well as also acting as DNase to break double-stranded DNA (dsDNA) (Wang et al., 2017; Zetsche et al., 2017; Zhang et al., 2017). The Cpf1 endonuclease consists of the RuvC domain, but it lacks the HNH domain, unlike Cas9 endonuclease (Yamano et al., 2016). The mutation within the RvuC domain of Cpf1 leads to loss of nuclease activity for both strands of target DNA (Zhang et al., 2017). The mutation at site E993A within the RuvC domain of AsCpf1 produces a DNAse-dead Cpf1 (ddAsCpf1) (Yamano et al., 2016; Zhang et al., 2017). ddAsCpf1-mediated multiplex gene regulation was demonstrated in *Escherichia coli (E. coli)* by targeting promoter region. ddAsCpf1 only—without any transcriptional regulator—is enough for targeted repression by its blocking of the transcription initiation and elongation of the desired gene (Zhang et al., 2017). Additionally, ddAsCpf1 can transcriptionally regulate multiple genes by delivering multiple crRNAs, which are subsequently cleaved by its RNase activity. The codon-optimized dead AsCpf1 (D908A) has efficiently been used in bacteria and plants as a transcriptional repressor (Tang et al., 2017; Zhang et al., 2017). In plants, dead Cpf1-mediated transcriptional repressor was successfully used in Arabidopsis and rice. Dead AsCpf1 and LbCpf1 were shown to be efficient for transcriptional repression, and results showed a 10-fold reduction of miR159b transcription (Tang et al., 2017). The transcriptional effector proteins can be fused with catalytically inactive (i.e., dead) Cpf1 and efficiently used in plant and animal systems in a manner similar to dead Cas9. Recently, KRAB, VP64, and VPR domain sequences were fused with dead AsCpf1 at the C terminus and were used for targeted repression and activation in human cells (Liu et al., 2017). In another study, a drug-inducible dead LbCpf1, along with transcriptional activators, was used to enhance the expression of multiple genes (Tak et al., 2017).

## CRISPR/Cpf1-Mediated Editing in Plants

CRISPR/Cpf1-mediated genome editing has more potential than other tools such as TALEN, ZnFN, and CRISPR/Cas9. The usefulness of CRISPR/Cpf1 has been successfully demonstrated for targeted mutagenesis in Arabidopsis, rice, tobacco (*Nicotiana tabacum*), soybean (*Glycine max*), maize (*Zea maize*), citrus (*Citrus X sinensis*), cotton (*Gossypium hirsutum*), etc. (Endo et al., 2016; Kim H. et al., 2017; Tang et al., 2017; Wang et al., 2017; Xu et al., 2017, 2019; Lee et al., 2019). We have tabulated recent works on the Cpf1-mediated gene editing in different plants species in Table 1. In a comparative study of rice, using CRISPR/Cpf1- and CRISPR/Cas9-mediated editing to knock out the *Epidermal Patterning Factor like-9* (*EPFL9*) gene, LbCpf1 showed a higher percentage of mutated T0 plants compared to Cas9. LbCpf1 showed a maximum deletion size of 63 bp, whereas Cas9 showed a maximum deletion size of 37 bp (Yin et al., 2017). Endo et al. (2016) showed mutation frequencies of 28.2 and 47.2% in tobacco and rice, respectively, using CRISPR/Cpf1 at the targeted regions of the genome. Specific and efficient gene insertion or replacement within a genome is also a need in plant genetic engineering. Both LbCpf1 and FnCpf1 endonucleases were used for targeted gene insertion via homology-directed recombination (HDR) in plants. In addition, the targeted insertion frequency for the Cpf1 nuclease was shown to be up to 8%, which is superior compared to other known genome editing endonucleases in rice (Begemann et al., 2017). The size of the gene encoding for *Cpf1* is smaller than that of *Cas9*, thus reducing the overall size of the plant transformation vector, which makes for easy packaging and transfer into plant cells.

**TABLE 1:** List of different application of CRISPR/Cpf1 mediated genome modification in plants.

Plant name	Transformation method	Binary vector	Selectable marker	Target genes	Reference
Rice	*Agrobacterium*	pCAMBIA	*hptII*	*EPFL9*	Yin et al., 2017
Rice	*Agrobacterium*	pPZP200	*hptII*	*DL* *ALS, NCED1* *AO1*	Endo et al., 2016
Rice	*Agrobacterium*	pHSN400	*hptII*	*OsPDS, OsBEL*	Xu et al., 2017
Rice (Multiplexing)	*Agrobacterium*	pCambia	*hptII*	*OsRLK, OsBEL*	Wang et al., 2017
Soybean, Tobacco	PEG-mediated protoplasts transformation	p2GW7	DAPI and Cy3 fluorophore probe	*FAD2-1A, FAD2-1B*	Kim H. et al., 2017
Tobacco	*Agrobacterium*	pRI201-AN	*nptII*	*STF1, NtPDS*	Endo et al., 2016
Allotetraploid cotton	*Agrobacterium*	*pGhRBE3−Cpf1−GhCLA1*	*NA*	*Cloroplastos alterados (GhCLA)*	Li et al., 2019
Maize	*Agrobacterium*	pYPQ141, 210, 230	*Bialaphos−resistant*	*Maize glossy2 gene*	Lee et al., 2019
Rice	Protoplasts transformation	STU−Cas12a system	*NA*	*OsDEP1* *OsROC5*	Tang et al., 2019
Rice	*Agrobacterium*	STU−poly−A vector	*NA*	*OsPDS* *OsDL*	Xu et al., 2019
Arabidopsis and rice	Floral dip and protoplasts transformation	dAsCpf1–SRDX and dLbCpf1–SRDX carrying vector	*NA*	*OsDEP1* *OsROC5* *OsPDS*	Tang et al., 2017
Arabidopsis	Floral dip	enAsCpf1 and ttLbCas12a carrying vector	*NA*		Schindele and Puchta, 2019
Rice	Protoplasts transformation	Pol II promoter and ribozyme processing system	*NA*	*DEP1, PDS, and EPFL9*	Zhong et al., 2018

*NA, not available.*

The vector-carrying construct of the Cpf1 protein along with *in vitro* synthesized CrRNA can be delivered into plant cells via *Agrobacterium*, bombardment, and PEG. DNA-free or vector-less editing using the CRISPR/Cpf1 complex has efficiently and frequently been carried out in mammalian cells and can also be performed in plants using PEG-mediated protoplast transformation (Figure 1). In this method, purified Cpf1 proteins, along with *in vitro* synthesized guide RNA, was transferred into animal cells via PEG or microinjection (Moreno-Mateos et al., 2017). Cpf1 proteins, along with guide RNA complexes, are used as alternative ways for genome editing of plants without introducing *DNA* into plant cells, and thus referred to as a DNA-free editing system. Recently, Cpf1 proteins and gRNA were efficiently delivered to soybean and wild tobacco protoplasts without T-DNA integration (Kim H. et al., 2017).

## Multiplex Editing Using CRISPR/Cpf1

**FIGURE 2:**
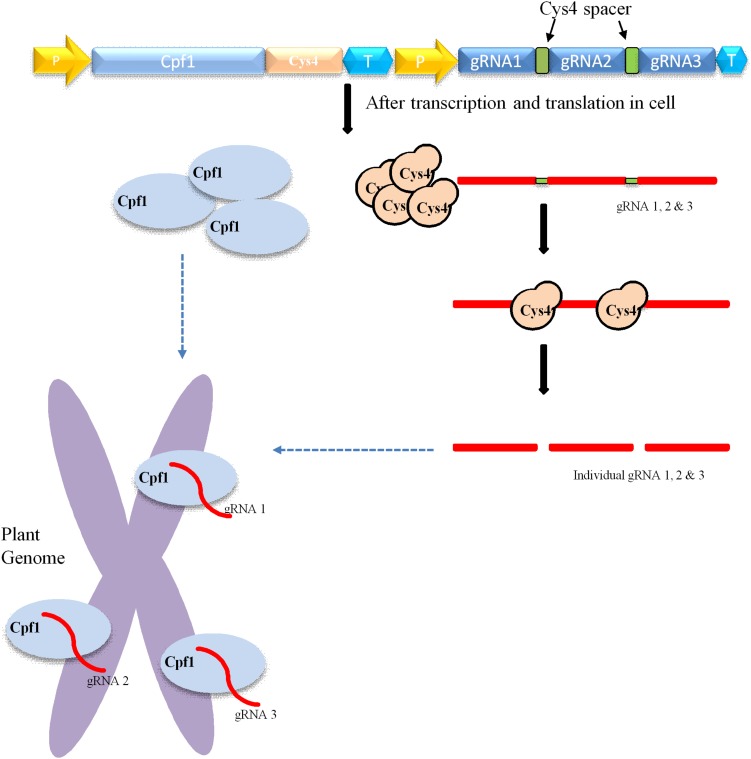
Mechanism of Cpf1-cys4-mediated multiplex genome editing. Construct carrying Cpf1, CRISPR system *Yersinia* (Csy4), and various gRNAs. The yellow arrow indicates the promoter; the *Cpf1* gene is indicated by sky blue; T means the terminator; and green square boxes represent Csy4 spacers. The delivery and integration of construct into the plant genome leads to the transcription and translation of Cpf1 (sky blue oval) and Csy4 endonuclease (brown circle) within the cell. Csy4 endonuclease acts on spacers (green) and separate individual gRNA1, 2, 3, and so on. Furthermore, these individuals gRNAs bind to target their respective sites within the plant genome, where Cpf1 endonuclease creates double-stranded breaks.

**FIGURE 3:**
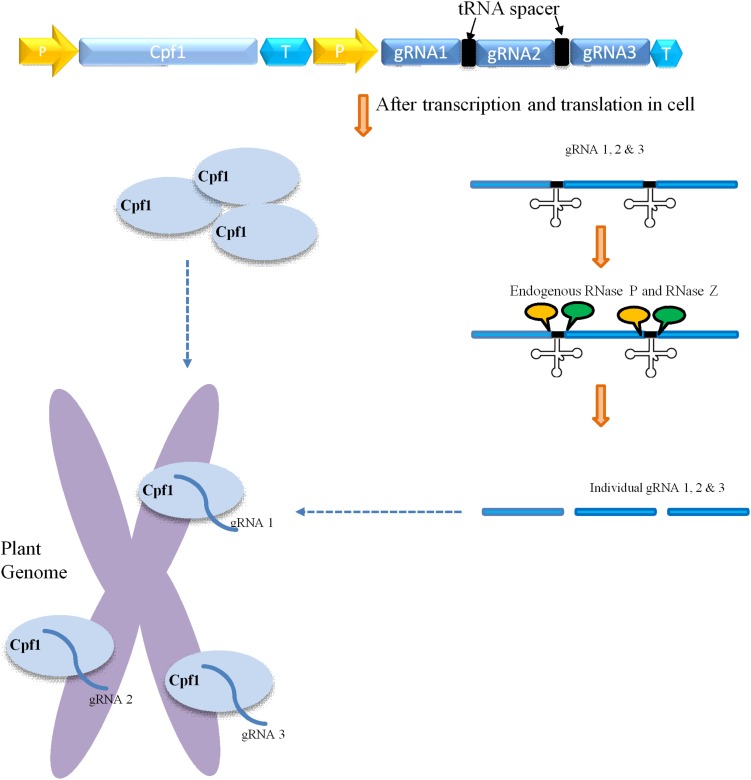
Mechanism of Cpf1-glytRNA-mediated multiplex genome editing. The construct consists of the Cpf1 gene and multiple tRNA-gRNA units. Each gRNA (blue rectangle) containing a plant-specific tRNA spacer (black color). The delivery and integration of construct into plant genome leads to expression of Cpf1 and tRNA-gRNA primary transcript. The primary transcript carries gRNA along with tRNA (black cloverleaf structure), which is cleaved by endogenous RNase P and RNase Z (labeled as the yellow and green circle, respectively). The separated individual gRNAs direct Cpf1 to their respective targets within the plant genome.

CRISPR/Cas9-mediated multiplexing has been extensively applied for the alteration of numerous loci in plant genomes. Generally, this multiplexing is performed using two methods. The first one involves expressing many single gRNAs under different small RNA promoters either in same vector or in different vectors. In the second method, multiple single gRNAs are fused with a tRNA recognition sequence, which are expressed as a single transcript under one promoter (Figure 3). Furthermore, these multiple gRNAs are separated into individual gRNAs by endogenous ribonucleases of plant cells (Xie et al., 2015; Tang et al., 2019). In another strategy, the Csy4 gene is expressed with the Cas9 and the Csy4 recognition sequence are fused as a spacer between multiple guide RNAs (Figure 2). The dual activity of Cpf1, cleaving target DNA as well as cleaving its own crRNA, makes it suitable and the easiest way for multiplexing than Cas9 (Zetsche et al., 2015; Fonfara et al., 2016). Unlike Cas9, Cpf1 does not need the support of tracrRNA during maturation of crRNA. Considering these benefits, Cpf1 has been accepted for the editing of multiple genes using a single crRNA array spaced by mature direct repeats in mammals and plants (Zetsche et al., 2015; Wang et al., 2017).

## CRISPR/Cpf1 Increased Targeted and Precise Gene Insertion

Gene insertion at a desired and specific position within a plant genome is in demand in plant genetic engineering for the purpose of developing crops with specific traits. This is generally achieved by the well-known plant repair mechanism, i.e., homology-directed repair (HDR). Another repair mechanism—non-homologous end-joining repair (NHEJ)—occurs in plants that generate indel mutations. In the somatic cells of plants, double-stranded breaks are efficiently repaired by NHEJ, which dominates over the HDR (Jiang et al., 2013). Generally, NHEJ results in diverse types of mutations, such as insertion, deletion of few base pairs, chromosome rearrangement, and chromosome relocation. HDR is the key method to carry out precise gene insertion; however, it is limited due to its lower efficiency (Moreno-Mateos et al., 2017).

In past few years, Cas9-mediated gene insertion has been successfully carried out; the insertion rate was 2.5–4.1 and 2.0–2.2% in maize and rice, respectively. However, the targeted gene insertion in rice using FnCpf1 and LbCpf1 endonucleases was achieved up to 8% (Begemann et al., 2017; Li et al., 2018a). The indels generated by Cas9 inhibit recurrent cleavage due to mismatches created within the seed region of the target DNA, whereas Cpf1 can repeat cleavages because it cuts ∼18 nucleotides away from the PAM. Due to this nature of Cpf1, it may boost the chance to repair a double-stranded break through HDR (Moreno-Mateos et al., 2017). The rice *Chlorophyllide-a oxygenase* (*CAO1*) gene, which converts chlorophyll a to chlorophyll b, was targeted in rice for gene insertion (Begemann et al., 2017). The disruption of the *CAO1* gene leads to yellowing of plants, which was used as a visual marker for gene insertion. The CRISPR/Cpf1 vector and donor template plasmid were co-transformed in rice embryogenic calli using the bombardment method (Begemann et al., 2017).

## Cpf1-Mediated Base Editing in Genome

DNA base editing requires chimeric protein comprised of an RNA-guided endonuclease and an enzyme able to deaminate an adenine or a cytidine base. The fused deaminating enzyme may be cytidine deaminase (C-to-T) or adenine deaminase (A into G or A–T into G–C). APOBEC (apolipoprotein B mRNA-editing enzyme catalytic polypeptide–like) and AID (activation-induced deaminase) have been used as cytidine deaminase frequently. Initially, base editing was performed in plants with Cas9 that fused a different deaminating domain (Shimatani et al., 2017). However, due to a limitation of the Cas9 PAM site, Cpf1-mediated base editors are in demand and can target T-rich sequences of a plant genome. The first Cpf1-mediated cytidine deaminase base edit was done in human cells in Li et al. (2018c). Li and colleagues generated a Cpf1-mediated base editor by fusing a rat APOBEC1 domain and uracil DNA glycosylase inhibitor to dLbCpf1. They named this base editor dLbCpf1-BE0, and it was effective for sites, T-rich regions, where Cas9 could not bind (Li et al., 2018c).

The dLbCpf1-BE0 showed an editing effect from position eight to 13 bp preceding the PAM (the base next to the PAM was counted as position one). Furthermore, dLbCpf1-BE0 showed low levels of unintended indel mutations and non-C-to-T substitutions with a 20–22% base editing efficiency (Li et al., 2018c). An enhanced *Acidaminococcus* sp. Cpf1 variant (also known as enAsCas12a) increased the base editing range with altered PAM in human cells (Kleinstiver et al., 2019). However, to date there has been no report of Cpf1-mediated base editing in plants (Malzahn et al., 2019).

## Cpf1 Features and Limitations

Cpf1 endonuclease is small in size and consists of only RuvC-like endonuclease domains in contrast to Cas9 endonuclease, which consists of RuvC-like and HNH domains. CRISPR/Cpf1 cleaves both strands of target DNAs in a staggered pattern, whereas Cas9 cleaves blunt ends. Cas9 requires multiple Pol III promoter to drive various gRNA, whereas Cpf1 needs only one promoter to regulate several crRNA (Zetsche et al., 2017). Cpf1 endonuclease requires only 42–44-nucleotide-long crRNA instead of the engineered 100-nucleotide-long gRNA (tracrRNA and crRNA) desired by Cas9 endonuclease. CRISPR/Cas9 cleaves DNA near PAM, while Cpf1 cleaves DNA distal to PAM without disrupting the 19 bp target sequences. Therefore, Cpf1 gives opportunities for future modifications at the same target site. This suggests that CRISPR/Cpf1-mediated editing may improve the frequency of HDR over NHEJ. Cpf1 endonuclease mediated editing give less errors with controllable insertions due to its sticky ends. Various other features make it more suitable for genome modification than Cas9, and these are listed in Table 2.

**TABLE 2:** Different features of Cpf1 and Cas9 mediated editing.

Features	CRISPR/Cpf1	CRISPR/Cas9
Classification	Class 2, Type II system	Class 2, Type V system
gRNA	75 bp chimeric of tracrRNA and CrRNA	44 bp CrRNA
PAM recognition sequence	5′-TTTV-3′	5′-NGG-3′
PAM	T-rich PAM site	G-rich PAM site
Cleavage	Distal to PAM site	Proximal to PAM site
Molecular weight	157,900 g/mol	163,700 g/mol

There are a limitation to Cpf1, and these include shorter crRNA as well as specific temperature requirements during plant genetic transformation (Bernabé-Orts et al., 2019; Lee et al., 2019; Malzahn et al., 2019; Schindele and Puchta, 2019). The crRNAs for Cpf1 are shorter than Cas9, which may lead to the formation of undesirable secondary structures and decreased efficiency, as shown in case of maize (Lee et al., 2019; Malzahn et al., 2019). The maize glossy2 gene was targeted by SpCas9 and LbCpf1 for *Agrobacterium*-mediated genome editing. Results showed 90%–100% editing efficiency in the case of Cas9-edited T0 plants, whereas there was 0%–60% editing efficiency in the case of Cpf1-edited T0 plants.

Cpf1-facilitated genome alteration is temperature sensitive in plants, and this is a major limitation (Lee et al., 2019; Malzahn et al., 2019). The effects of low temperature on the activity of AsCpf1, FnCpf1, and LbCpf1 were documented in Arabidopsis, rice, and maize (Malzahn et al., 2019). For better editing efficiency AsCpf1 requires high temperatures of 28°C or above, and LbCpf1 showed very low efficiency at 22°C that reached to 100% at 28°C. A variant of AsCpf1, enhanced AsCpf1 or enAsCpf1, showed 2-fold higher activity at lower temperature in human cells (Kleinstiver et al., 2019). A comparison between LbCpf1, Enhanced LbCpf1 (enLbCpf1), and temperature-tolerant LbCpf1 (ttLbCpf1) was conducted at low temperature (22°C) in Arabidopsis. The result showed that, out of these three, ttLbCpf1 activity was highest at 22°C (Schindele and Puchta, 2019).

## Conclusion

In this review, we discussed CRISPR/Cpf1 characteristics and its rise to prominence as a powerful genome-editing tool for plants. The Cpf1 orthologous reported from various bacteria and the PAM site variation were significantly useful since they increased the flexibility of the target choice within the genome. Unique features of Cpf1 make it suitable for various applications across diverse plants species, more so than Cas9. Furthermore, CRISPR/Cpf1-mediated knock-in of desired genes showed increased targeted gene insertion. Moreover, the smaller size of Cpf1 may allow for a non-transgenic route, such as viral vector mediated delivery of the CRSIPR/Cpf1, of the genome modification. The potential of the CRISPR/Cpf1 system in trait improvement without a transgene is a well-sought-after approach for the community.

## Author Contributions

AA conceptualization the idea. DS, PJ, and AA wrote the manuscript. VR, KB, and JK reviewed and edited. HS drew the image and tabulated the table.

## Conflict of Interest

The authors declare that the research was conducted in the absence of any commercial or financial relationships that could be construed as a potential conflict of interest.

## Footnotes

1 http://www.rgenome.net/cpf1-database/
